# Influence of Two Types of Guide Harnesses on Ground Reaction Forces and Step Length of Guide Dogs for the Blind

**DOI:** 10.3390/ani12182453

**Published:** 2022-09-16

**Authors:** Anna Weissenbacher, Alexander Tichy, Karl Weissenbacher, Barbara Bockstahler

**Affiliations:** 1 Section of Physical Therapy, Small Animal Surgery, University Clinic for Small Animals, Department of Companion Animals and Horses, University of Veterinary Medicine, 1210 Vienna, Austria; 2 Platform Bioinformatics and Biostatistics, Department of Biomedical Sciences, University of Veterinary Medicine Vienna, 1210 Vienna, Austria; 3 Testing and Coordination Centre for Assistance Dogs, Messerli Research Institute Department of Interdisciplinary Life Sciences, University of Veterinary Medicine Vienna, 1210 Vienna, Austria

**Keywords:** guide dog, harness, ground reaction forces, step length, biomechanics

## Abstract

**Simple Summary:**

Guide dogs for the blind are an important tool for their handlers to live an independent life. As previous studies have shown, the harness has a significant impact on the dog’s kinematics and exerts pressure on special areas of the dog’s body. The aim of the study was to evaluate the influence of two types of harness on the force distribution between the paws. To measure the influence of the harnesses, we compared the vertical ground reaction forces in the working harness of a guide dog (Norwegian type) and in a Y-harness, each with a leash, a straight handle and a handle bent on the left side. Furthermore, the ground reaction forces were measured in dogs with collar and leash. Twelve certified guide dogs were included in the study. Compared to walking with collar and leash, none of the harnesses, when used with a leash, had an effect on the evaluated parameters. When the dogs walked in the harness with a handle and, as is common when leading blind people, under a light pull, there were clear effects on the impulse. Future studies should be devoted to the type of attachment of the harness, as well as the angle of attachment, which is altered by the size of the handler.

**Abstract:**

Few studies exist addressing the effects of guide dog harnesses on dogs biomechanics. The aim of this study was to investigate how two different harness types affect ground reaction forces and stride length. Twelve certified guide dogs were tested under different conditions: walking with a collar and leash, walking with the harness used daily (Norwegian type with straight handle) and walking with a Y-harness using a straight or a curved handle. The parameters studied included maximum vertical force, vertical impulse and stride length. Compared to walking with a collar and leash, none of the harnesses, when used with a leash, had an effect on the evaluated parameters. However, both harnesses, when used with a handle and under re-enactment of the lead work, showed clear effects on the impulse. Stride length was shortened if the Y-harness with handles was used. Future studies should focus on the type of attachment of the harness, as well as the angle of attachment, which is altered by the size of the handler. The development of individually adapted harnesses in order to subject these animals to as little stress as possible during their daily work should be one of the future areas of research.

## 1. Introduction

Guide dogs have been accompanying blind and visually impaired people for hundreds of years [1]. They guide blind and visually impaired people safely through road traffic and, thus, make an important contribution to their independence. Although there are already numerous technical aids, guide dog handlers prefer their dogs to these aids [2]. Many guide dogs are also considered part of the family [3]. For the aforementioned reasons, and also due to the expensive and time-consuming training of a guide dog, it is important that a guide dog can complete a long and, above all, healthy service. To ensure this, the dogs’ musculoskeletal system must also be kept healthy. 

The influence of the harness on the biomechanics of the dogs has aroused interest. In principle, a distinction can be made between different types of harnesses, the best known of which are the Y-harness and the so-called Norwegian harness. In the former, a strap runs along the sternum, divides over the chest aperture, crosses the shoulder joints and rejoins at the back and is then connected to the waist strap. In the Norwegian type, a strap runs horizontally along the chest, along the side of the shoulder and is then connected to the abdominal belt on the right and left sides.

There are only a few studies on the subject. Galla et al. [4] studied the influence of three different guide harnesses (Norwegian type) on the spinal movement of guide dogs. The first harness was made of leather and had a back strap, a chest strap running laterally, a strap running across the chest apex and an additional strap between the forelimbs. The handle was fastened with the help of carabiners and was limited in movement by loops. The second harness consisted of a padded back strap, a strap running across the chest apex and a leather chest strap running along the sides. The handle was attached with the help of two quick-release buckles made of hard plastic. The third harness was similar to the second harness, but it lacked the padding on the back strap and the side and front chest straps were each adjustable in size with the help of Velcro fasteners. The handle of this harness was bent to the left and connected to the harness by two metal fasteners with springs. Eight adult, healthy, trained guide dogs of different breeds participated in the study. Reflective markers were placed on the dogs’ heads, along the spine at C7, Th13 and S3, and on the left forelimb, lateral to the distal metacarpus. The dogs led a handler through three different exercises: walking straight ahead, a turn to the left and a turn to the right. Each exercise was performed with each of the three guide harness models. Markers C7-Th13-S3 were used to calculate the angles of the spine in the dorso-ventral and latero-lateral directions. Harnesses 1 and 3 showed a restriction in the range of spinal motion in the dorso-ventral direction when walking around an obstacle to the right compared with walking in a straight line. Harness 1 showed a restriction of spinal motion when walking in a straight line compared to walking in a straight line without a harness. There was a similar result when walking around an obstacle to the left. Harness 1 thus caused a significant reduction in spinal motion in the latero-lateral direction during all three exercises, while the other two harness types did not demonstrate this limitation.

In the study by Peham et al. [5], the pressure distribution under the harness (using pressure stripes attached to the harnesses) of guide dogs was investigated. The same dogs, guide harnesses and exercises were used in this study as in the study by Galla et al. [4]. For all harnesses, the highest pressure was found in the right sternal region, but the back region showed little load. The different harnesses had a significant effect on the pressure, while the exercises had little effect. The third harness, with the hard plastic fasteners, showed less load on the sternum in all exercises compared to the other two harnesses.

Lafuente et al. [6] tested the influence of a Y-harness (“non-restrictive”) and a Norwegian (“restrictive”) harness on the extension of the shoulder at walk and trot. Nine dogs participated in the study. Non-reflective markers were placed on the dogs on the left, proximal to the scapula on the spine; on the acromion; on the lateral humeral epicondyle; and on the ulnar styloid process. These markers served as landmarks to calculate the angle of the shoulder joint in maximum extension. The measurements were performed on a treadmill to ensure a constant speed. The dogs were tested in five different situations, which were always performed in the following order: no harness, Y-harness without weight, Y-harness with weight attached, Norwegian harness without weight and Norwegian harness with weight attached. The weight was to simulate pulling or work. This was carried out with the help of a leash that first passed through the 2.5 kg weight, then through a ring on the upper part of the frame of the treadmill, diagonally above the dog, and finally attached to the D-ring of the harness on the dog’s back. Each situation was filmed for 30 s, as 12 angle measurements were needed for each dog in each situation to reduce errors and measurement inaccuracies. They found that the Y-harness and the Norwegian harness significantly restricted shoulder extension. In their study, the Norwegian harness restricted shoulder extension less than the Y-harness. With the Y-harness, they were able to measure a reduction of around 5° at a walk and around 9° at a trot, compared to around 2° at a walk and around 5° at a trot with the Norwegian harness. The Y-harness with weight restricted the extension of the shoulder significantly more than the Y-harness without weight. This was not found with the Norwegian harness.

All the studies mentioned have provided important information on the kinematics of joints and pressure distribution under harnesses. So far, the influence of different types of harnesses on the ground reaction forces (GRFs) of dogs has not been investigated. However, this is a relatively simple way to show the influence of harnesses on the forces acting on the extremities.

The aim of the study was therefore to investigate the ground reaction forces of healthy guide dogs when wearing their own harness (Norwegian type and straight handle), their own harness with a curved handle and a Y-harness with a straight and curved handle. The hypotheses were that both the Y-harness and Norwegian-type harnesses would have a significant effect on the ground reaction forces and stride length and that a curved handle would have a lesser effect than a straight handle.

## 2. Materials and Methods

The study was reviewed and approved by the Ethics and Animal Welfare Committee of the University of Veterinary Medicine Vienna with regard to its compliance with Good Scientific Practice and relevant national legislation (Reference ETK-032/02/2020). The guide dog handlers were informed about the procedure of the measurements and, subsequently, signed the consent form. 

### 2.1. Dogs

For this study, 12 guide dogs that had already passed the official test according to § 39a of the Federal Disability Act of the Republic of Austria were recruited. An inclusion criterion was that the dogs had to be led in a Norwegian harness with a straight handle. Furthermore, the dogs were not allowed to exhibit abnormalities in the orthopedic examination and had to have an SI of <3% (see Section 2.4). Nine Labrador retrievers, one flat-coated retriever, one curly-coated retriever and one large poodle participated in the study. The dogs ranged in age from two to eight years, with a mean ± standard deviation (SD) of 5.0 ± 2.2 years, and in body mass from 23 kg to 39.3 kg, with a mean ± SD of 31.6 ± 4.2 kg. Nine male neutered and three female neutered dogs participated in this study. 

### 2.2. Harnesses and Handles

**Figure 1:**
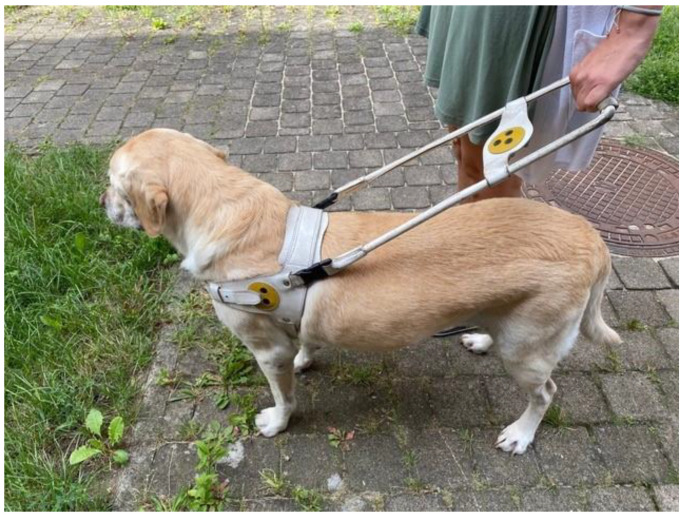
Standard Norwegian harness. Example of a usual working harness from dog 5.

**Figure 2:**
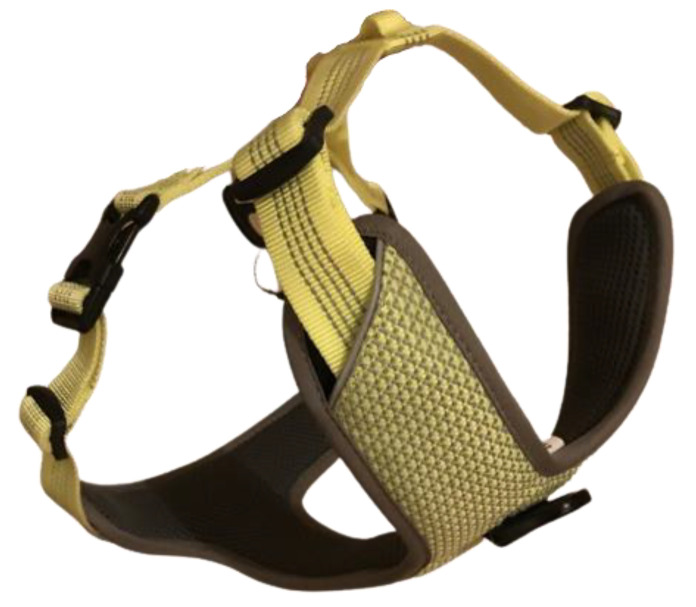
Y-harness (Joshua Reflective functional dog harness, Sugar Dog, Krämer Pferdesport, Germany).

**Figure 3:**
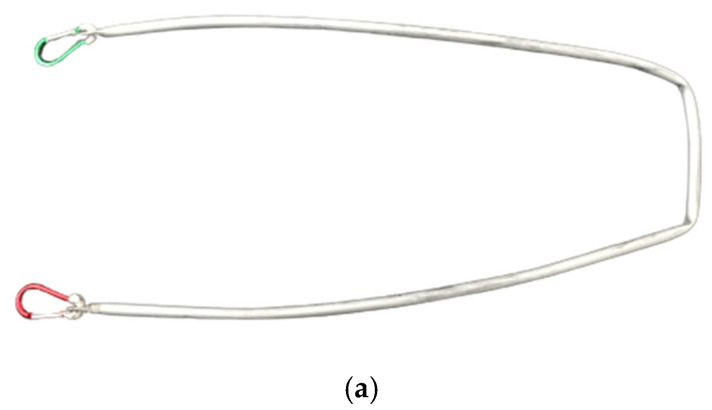

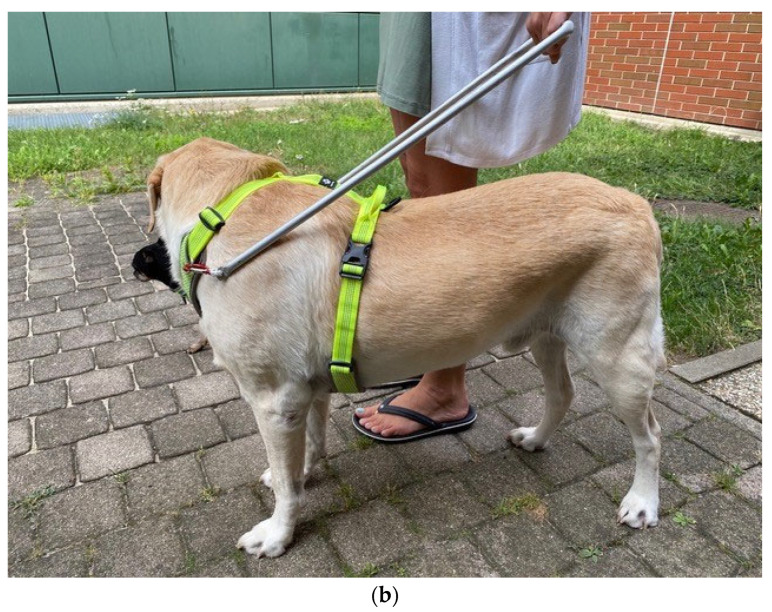
(**a**) Straight handle (Realprojekt Metallbau, Austria). (**b**) Y-harness with straight handle (dog 5).

**Figure 4:**
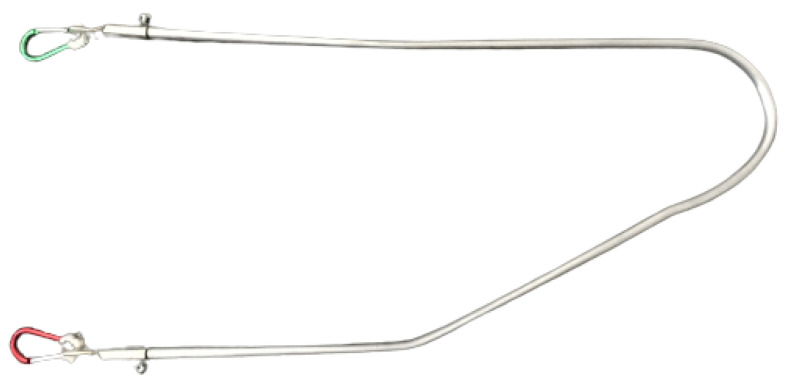

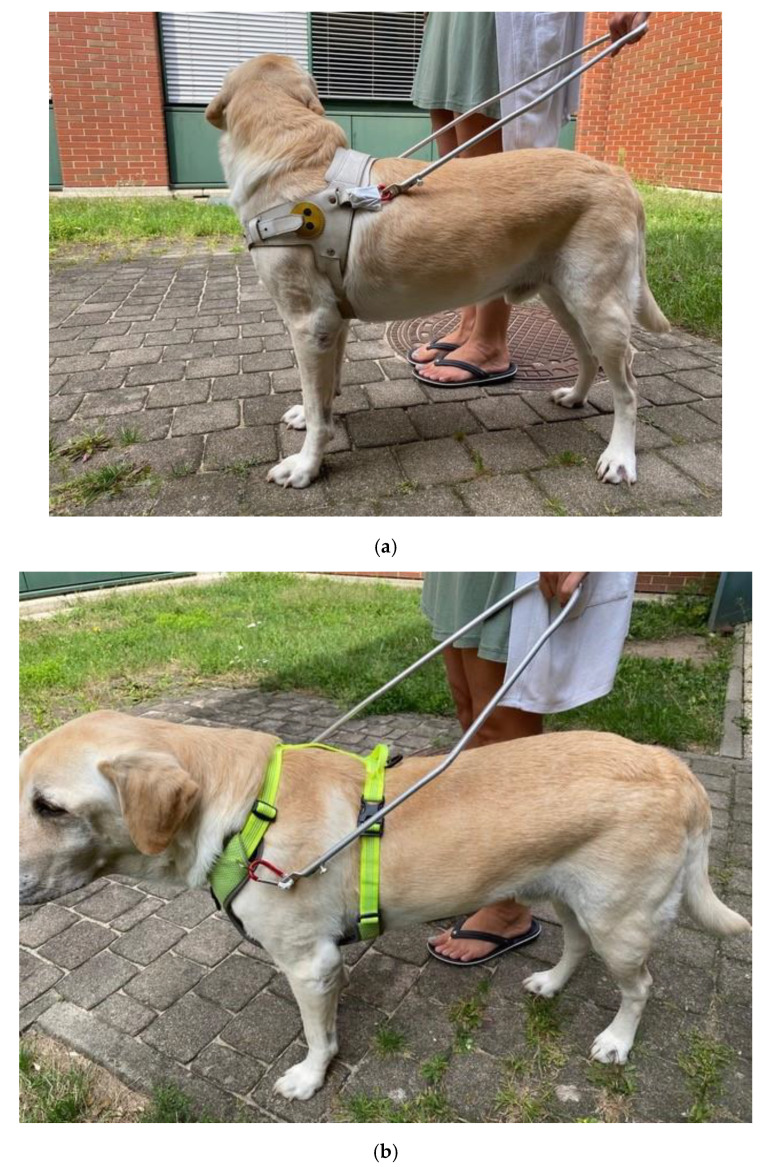
Handle, bent on the left side (Realprojekt Metallbau, Austria). (**a**) Standard Norwegian harness with the curved handle. Example of the usual working harness from Dog 5. (**b**) Y-harness with curved handle (dog 5).

The following equipment was used:

- The harness and the handle that the dogs wore in their daily life during the guide work. All harnesses were of the Norwegian type, and the handle was a straight handle (Figure 1);

- A Y-harness (Joshua Reflective functional dog harness, Sugar Dog, Krämer Pferdesport, Germany; Figure 2);

- A straight handle (Realprojekt Metallbau, Austria; Figure 3—Figure 3a shows the Y-harness with the straight handle);

- A curved handle (Realprojekt Metallbau, Austria; Figure 4—Figure 4a shows a Norwegian harness with a curved handle, Figure 4b the Y-harness with a curved handle).

### 2.3. Measurement Procedure

Measurements were recorded using a Zebris FDM type 2 pressure plate (Zebris Medical GmbH, Allgäu; Germany). The pressure plate had a measuring area of 203.2 cm × 54.2 cm, 15,360 sensors and a scanning frequency of 100 Hz. This was integrated into the floor, so there was no difference in level between the floor and the plate. Both the plate and surrounding floor were covered with 1 mm thick pond liner so as to have no visual or haptic difference between the floor and plate. Furthermore, the passing of the pressure measurement plate was filmed using a camera placed diagonally behind the pressure measurement plate. This served as an aid to match the paws to the data. Before the measurements, the dogs were given the opportunity to move freely in the room and explore it. The Y-harness was fitted to the dogs so that the neck straps were not above the shoulder joint and there were at least two finger widths of space between the belly strap and the front limb. The dogs were then observed to ensure that they could move comfortably in the harness. Afterwards, the dogs were led back and forth in the harness several times until they showed a smooth gait pattern, and only then was measurement started. For the measurements, all dogs were walked by the same sighted person (AW, body height 174 cm). Since the dogs are trained to walk on the left side of the handler, they were also led on the left side for the measurements. The order of measurements was determined by randomization. A measurement was counted as valid if the dog looked forward; did not overstride; walked at a steady pace; did not pull on the leash in conditions 1, 2 and 5; and brought a constant light pull on the handle in C3, C4, C6 and C7. At least five valid trials were used for the further evaluation. 

Ground reaction forces were measured in the following conditions (C) in randomized order:

- C1: Walking with collar and leash;
- C2: Usual working harness of the dog (Norwegian type) with leash;
- C3: Usual working harness of the dog with usual working handle (straight handle);
- C4: Usual working harness of the dog with curved handle;
- C5: Y-harness with leash;
- C6: Y-harness with straight handle connected to the harness with a carabiner;
- C7: Y-harness with curved handle connected to the harness with a carabiner.

### 2.4. Data Analysis

The collected data were analyzed using special software (Pressure Analyser 4.6.3.0, Michael Schwanda). The following parameters were used for evaluation:

- Peak vertical force (pForce, N): this value was normalized to the total force and expressed as %TF using the following formula;

pForceLF (%TF) = pForceLF/(pForceLF+pForceRF+pForceLH+pForceRH)*100;

where pForce = peak vertical force (N), pForceLF = pForce (N) of the left front limb, pForceRF = pForce (N) of the right front limb, pForceLH = pForce (N) of the left hind limb, pForceRH = pForce (N) of the right hind limb;

- Time to pForce (TpForce): this parameter described the time at which pForce was reached and was expressed as a percentage of the stance phase duration (TpForce %SPD);
- Vertical impulse (vImpuls, Ns): this value was normalized as was described for pForce and expressed as vImpuls (%TF).

A symmetry index (SI%) for the front and the hind limbs was calculated for pForce and vImpuls. The following formula was used for this purpose:

SXFz (%) =abs ((XFzL−XFzR)/(XFzL+XFzR))*100;

where XFz represents pForce or vImpuls, L = left limb, R = right limb.

- Stance phase duration (s, SPD): this value was normalized as was described for pForce for the total stance time and expressed as the SPD %;
- Step length (SL, m);
- Velocity and acceleration: these parameters were evaluated for the left front leg to control the velocity and acceleration between the different measurement conditions. The differences in the velocity at which the dogs crossed the plate were within a range of ±0.3 m/s at a walk [7], with a maximum of 0.5 m/s at a trot [8], and they had an acceleration of ±0.5 m/s^2^.

### 2.5. Statistical Methods

Statistical analysis was performed with IBM SPSS statistics version 28. A normal distribution of the differences was assumed, which was checked with the Shapiro–Wilk test. We defined the C1 versus C2–C7 comparison as our primary outcome. Data were analyzed using a general linear model (GLM) and post hoc tests, with the harness and bracket factors included in the model as repeated-measures factors. For all statistical comparisons, a significance level of 5% (*p* ≤ 0.05) was considered significant.

## 3. Results

Velocity and acceleration did not differ between measurement conditions.

### 3.1. Norwegian Harness

**Figure 5:**
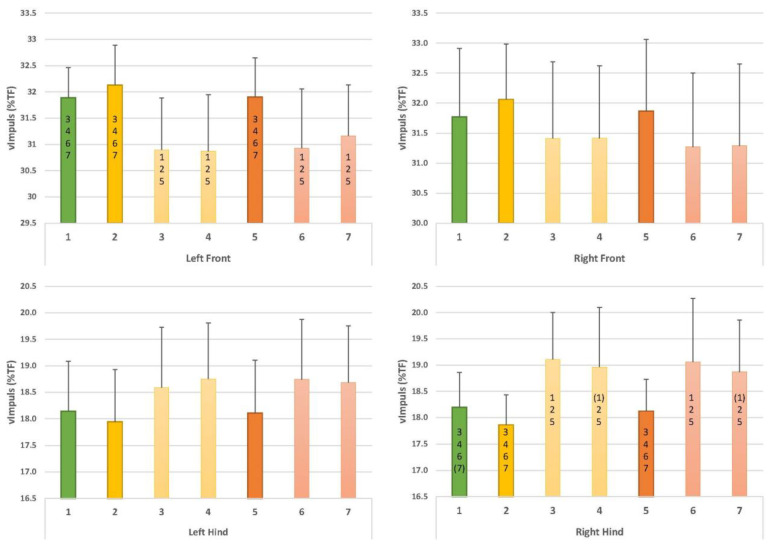
Results for vImpuls (%TF). Numbers on the x-axis denote conditions C1–C7. Green bar: collar and leash (C1). Yellow: usual working harness of the dog (Norwegian type) with leash (C2); with usual working handle (straight handle, C3); with curved handle (C4). Orange: Y-harness with leash (C5); with straight handle (C6); with curved handle (C7). Numbers within bars denote significant differences between conditions, numbers in parentheses indicate a *p*-value of 0.06. For example, in C4, vImpuls (%TF) in the left front limb was significantly different from C1, C2 and C5.

**Figure 6:**
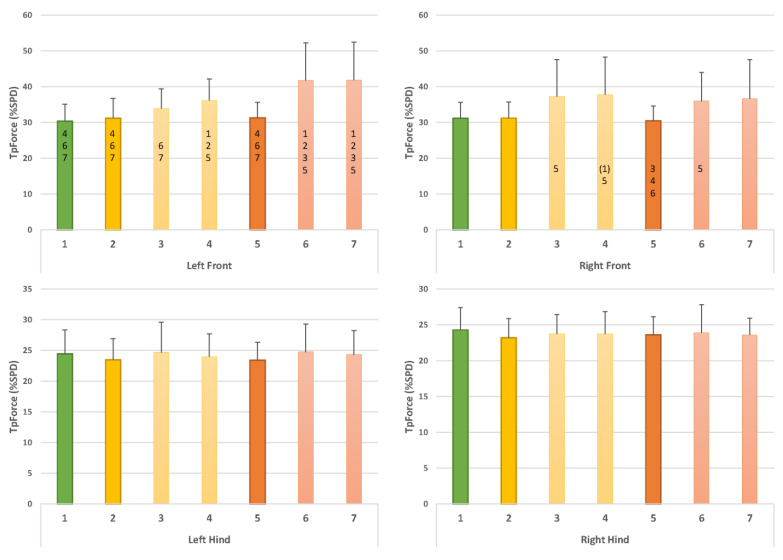
Results for TpForce (%SPD). Numbers on the x-axis denote conditions C1–C7. Green bar: collar and leash (C1). Yellow: usual working harness of the dog (Norwegian type) with leash (C2); with usual working handle (straight handle, C3); with curved handle (C4). Orange: Y-harness with leash (C5); with straight handle (C6); with curved handle (C7). Numbers within bars denote significant differences between conditions, numbers in parentheses indicate a *p*-value of 0.06. For example, in C4, TpForce (%SPD) in the left front limb was significantly different from C1, C2 and C5.

**Figure 7:**
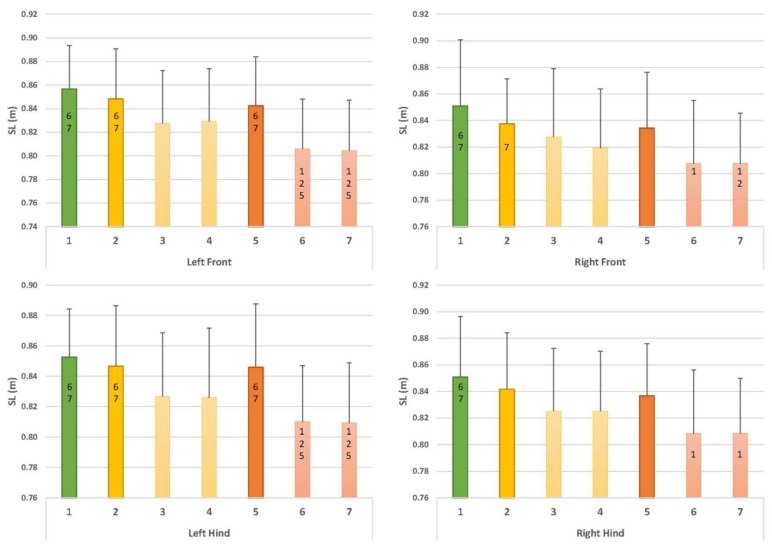
Results for SL (m). Numbers on the x-axis denote conditions C1–C7. Green bar: collar and leash (C1). Yellow: usual working harness of the dog (Norwegian type) with leash (C2); with usual working handle (straight handle, C3); with curved handle (C4). Orange: Y-harness with leash (C5); with straight handle (C6); with curved handle (C7). Numbers within bars denote significant differences between conditions. For example, in C6, SL (m) in the left front limb was significantly different from C1, C2 and C5.

- Comparisons with walking with collar and leash (C1) (Figure 5, Figure 6 and Figure 7):
  ○ Compared to walking with a collar and leash (C1), walking in this harness using a leash (C2) did not result in any significant changes in the parameters;
  ○ Leading with a straight handle (C3) resulted in a decreased vImpuls (%TF) at the front left (*p* = 0.01) and an increased vImpuls (%TF) at the hind right (*p* = 0.01);
  ○ Leading with a curved handle (C4) led to a reduced vImpuls (%TF) at the front left (*p* = 0.01); this value again appeared increased at the hind right but just outside the significance level (*p* = 0.06). Furthermore, TpForce (%SPD) appeared later at the front left (*p* = 0.02), and TpForce (%SPD) appeared later at the front right, just outside the significance level (*p* = 0.06).
- Comparison with harness with leash (C2) (Figure 5, Figure 6 and Figure 7):
  ○ Leading with a straight handle (C3) resulted in a decreased vImpuls (%TF) at the front left (*p* = 0.00) and an increased vImpuls (%TF) at the hind right (*p* = 0.00);
  ○ Leading with a straight handle (C4) resulted in a decreased vImpuls (%TF) at the front left (*p* = 0.00) and an increased vImpuls (%TF) at the hind right (*p* = 0.01).
- Comparison of straight handle versus curved handle (C3, C4) (Figure 5, Figure 6 and Figure 7):
  ○ None of the parameters showed significant differences in the direct comparison of the harnesses.

### 3.2. Y-Harness

- Comparisons with walking with collar and leash (C1) (Figure 5, Figure 6 and Figure 7):
  ○ Compared to walking with a collar and leash (C1), walking in this harness with a leash (C5) did not result in significant changes in the parameters;
  ○ Leading with a straight handle (C6) resulted in decreased vImpuls (%TF) at the front left (*p* = 0.02) and increased vImpuls (%TF) at the rear right (*p* = 0.04). TpForce (%SPD) occurred later in the front left (*p* = 0.00). The stride length of all four legs was significantly shortened (front left *p* = 0.00, front right *p* = 0.04, rear left *p* = 0.01 and rear right *p* = 0.04);
  ○ Leading with a bent handle (C7) led to a reduced vImpuls (%TF) at the front left (*p* = 0.01); this value again appeared increased at the hind right but just outside the significance (*p* = 0.06). Moreover, TpForce (%SPD) occurred later in the left front (*p* = 0.00). The stride length of all four legs was significantly shortened (left front *p* = 0.00, right front *p* = 0.03, left hind *p* = 0.01 and right hind *p* = 0.00).
- Comparison with harness with leash (C5) (Figure 5, Figure 6 and Figure 7):
  ○ Leading with straight handle (C6) resulted in decreased vImpuls (%TF) in the front left (*p* = 0.02) and increased vImpuls (%TF) in the hind right (*p* = 0.03). TpForce (%SPD) occurred later in the front left (*p* = 0.01) and front right (*p* = 0.05). Step length was shortened in the front left (*p* = 0.04) and hind left (*p* = 0.04);
  ○ Leading with bent handle (C7) resulted in decreased vImpuls (%TF) at the front left (*p* = 0.05) and increased vImpuls (%TF) at the hind right (*p* = 0.04). TpForce (%SPD) occurred later in the front left (*p* = 0.01). Step length was shortened in the left front (*p* = 0.04) and left hind (*p* = 0.04).
- Comparison of straight handle versus curved handle (C6, C7) (Figure 5, Figure 6 and Figure 7):
  ○ None of the parameters showed significant differences in the direct comparison of the harnesses.

### 3.3. Comparison of Norwegian Harness versus Y-Harness

○ There were no significant differences between conditions C2 and C5.
○ Y-harnesses (5, C6) showed the following changes compared to the Norwegian harness: compared to walking in the Norwegian harness and leash (C2), there was a significantly lower vImpuls (%TF) in the front left (C5 and C6: *p* = 0.01) and in the hind right, the value was higher than in C2 (C6 and C7: *p* = 0.01). TpForce (%SPD) occurred later in the front left (C6 and C7: *p* = 0.01). Step length was shortened in the front left (C6 and C7: *p* = 0.02), in the front right in C7 (*p* = 0.05) and in the hind left (C6 and C7: *p* = 0.03). Compared to C3, there was a later TpForce (%SPD) in the front left (C6: *p* = 0.03, C7: *p* = 0.04) (Figure 5, Figure 6 and Figure 7).

The descriptive statistics (means and standard deviations) are presented in Table 1.

## 4. Discussion

The results of this study partially support the hypotheses that both the Y-harness and Norwegian-type harnesses have a significant effect on the ground reaction forces and stride length, but they reject the assumption that a curved handle would have a lesser effect than a straight handle.

In order to test these hypotheses, a motion analysis was performed on a pressure measurement plate, which allowed the ground reaction forces to be calculated. Ground reaction forces describe the forces acting during the stance phase and, in principle, can be collected in three directions (vertical, medio-lateral and cranio-caudal), with vertical forces representing the largest component and, therefore, being frequently used in research [9]. The resulting forces are plotted over time and allow the calculation of certain parameters that allow an objective description of the acting forces [10]. Frequently used are the maximal vertical force and its temporal occurrence during the stance phase, as well as the vertical impulse, which can be represented by integrating the force over time. Accordingly, the impulse allows the description of the function over the entire stance phase [10]. Typically, in sound animals, there is little variation in these parameters between the contralateral limb pairs, whereas the front limbs experience higher forces than the hind limbs [11]. In addition to forces, temporal-spatial parameters, such as step length and stance phase duration, can also be calculated. Numerous studies have been devoted to the topic of lameness in dogs and the changes in ground reaction forces under particular loads. With reference to the results presented here, for example, it is of interest which compensatory changes in ground reaction forces occur in dogs with lameness of the front limbs. Here, it can be noted that, in the case of unilateral lameness, reduced GRFs occur in the affected limb and a compensation via the contralateral side takes place [12,13,14]; furthermore (but not always [12]), an increase in GRFs in the diagonal hind leg has been described at the same time [13,14]. 

The first important result is the finding that, in the comparison of walking with collar and leash versus walking in the harnesses on the leash, neither the Norwegian harness nor the Y-harness had an influence on the measured parameters. Furthermore, the stride length was not changed, a fact that does not fully correspond to the results found by Lafuente et al. [6], which describe a restriction of shoulder extension by harnesses of these designs. This may be because the reduction in shoulder extension was at a mean difference of about 4° (Y-harness) and 2° (Norwegian harness). It should also be considered that the measurements of Lafuente et al. were performed on a treadmill, which could have influenced the results. 

The results were completely different as soon as the dogs were led with a handle under light pull, as is usual in the daily work of guide dogs. In both harnesses, and regardless of the handle used, there was an increased vImpuls (%TF) in the hind right, compensated by a decreased vImpuls (%TF) in the front left. This situation is reminiscent of the compensatory effect of unilateral lameness described above [13,14]. However, since the dogs used here were orthopedically healthy, this result is more reflective of the function of the limbs—where the forelimbs behave in a similar manner to a compliant strut while the hindlimbs act in a similar manner to a lever [15,16,17]—and, consequently, reflects the traction work that the dogs have to perform. 

In addition, a delayed appearance of the pForce (TpForce %SPD) was observed when the animals worked in the Norwegian harness with curved handle and in the Y-harness with both handles. The pForce represents that point at which ground reaction forces reach their highest value and occurs in the first third of the stance phase when forward energy is decelerated. The reduced extension of the shoulder described by Lafuente et al. [6] could lead to this shift. Likewise, the greater effect that the Y-harness has on this parameter supports the findings of these authors, as they described a greater reduction in shoulder extension when wearing a Y-harness. Strikingly, the shortening of the stride length in all four legs was only seen when wearing a Y-harness with a handle, leading to the overall conclusion that a Y-harness has a greater effect on the dog’s biomechanics during lead work than a Norwegian harness. The limited stride length could be a result of the location of the handle attachment, so further studies should investigate whether a different attachment location (e.g., on the abdominal belt) also leads to this effect. Further, due to the positioning of the handler on the right side of the dog and pulling on the harness, there is, as shown by Peham et al. [5], a “leaning over” of the dog to the right (which leads to the increased pressure in the area “sternum right”), with simultaneous relief in the front left (reduced impulse) and a later occurrence of the TimepForce. One further factor that should be investigated in further studies is the influence of limb length on stride length when walking with different harnesses.

One drawback to this study is certainly that the dogs were all measured in their usual harness, while the Y-harness was the same for all dogs. Even if all harnesses were of the same type (Norwegian), we cannot judge minor differences in construction. Nevertheless, the results seem to be valid—the vImpuls (%TF) showed highly significant differences, while, except for the TpForce (%SPD) in the front left with curved handle, no other effects were observed. This contradicts our hypothesis that a curved handle would have less effects. This hypothesis was made because, as discussed previously [5], traction on a straight handle resulted in increased stress on the right sternal region and the rationale was that the “kink” in the handle would reduce this effect. However, in the present study, only the GRF was measured; pressure measurement under the harness was not possible. The latter, however, should be investigated in further studies, as this was the only situation in which a curved handle had some influence on the measurement results. Conversely, it must, of course, also be taken into account that the dogs were not familiar with the Y-harness from their daily work. Even if the dogs were given adequate time to become accustomed with the Y-harness, the influence of this fact cannot be excluded. However, this seems to be mitigated by the fact that a stronger influence of the Y-harness had also been described by Lafuente et al. [6]. However, all results must also be considered with respect to the low number of animals and the simultaneous high number of measurement conditions. A larger number of animals would increase the statistical power by implying an alpha correction. In this respect, the results of this study need to be repeated with much larger numbers of animals.

Overall, these results show that both harnesses have an influence on the biomechanics of the dogs, although to a different extent. This knowledge should be used to work on making harnesses that affect the dogs as little as possible. To date, however, there are no longitudinal studies that have investigated whether these biomechanical changes have an impact on the health of the animals. Although this knowledge is lacking, considering the fact that the training of dogs is very expensive and they are of enormous value to their owners, research should do its utmost to keep these valuable animals in good health as long as possible.

One factor that has not been investigated here is the way in which the handle is attached to the harness. A carabiner was used here to attach the straight and curved handle; another possibility would be a quick-release fastener, for example. It is quite conceivable that the connection between harness and handle influences the transmission of force. As the dogs were always led by the same person, the variability was reduced; however, this does not necessarily reflect the real situation. A different size ratio of human and dog and, therefore, a different angle of the handle can certainly also have an effect. A taller person will cause a steeper angle between the stirrup and the back of the same dog than a shorter person. The effects of the angle of pull—e.g., whether the stride length is influenced more or less by a steeper angle than by a flat angle—should be investigated in further studies. For this purpose, biomechanical models would also be useful, allowing a simulation of different combinations of body size for the handler in relation to the size of the dog.

In this respect, further studies should also be carried out. Another point that needs to be discussed is the fact that the dogs in this study were led by a sighted person. There is no question that this could have influenced the results. It must also be remembered that each blind person has a different guide style, so some blind people have a stronger pull on the handle than others. Furthermore, it should be considered that additional physical disabilities of the handler could be further influencing factors.

Furthermore, it should not be ignored that the conformation of the dogs (for example, the length of the legs, the width of the chest and similar factors) may contribute to the biomechanical effects. Future studies must be conducted to investigate this. Likewise, further, larger studies should investigate the influence of the dogs’ behavior. Each dog leads its owner a little differently, and even if this may be difficult to transfer into objective numbers, research should address this issue. Certainly, the way in which the dog is trained to the harness and the work with the human plays a role—therefore, this should be included in studies, not least to be able to give important hints to training centers.

All this would contribute to the goal of finding the ideal combination of harness and handle for each human/dog pair.

## 5. Conclusions

In summary, compared to walking with collar and leash, none of the harnesses, when used with leash, had an effect on the evaluated parameters. However, both harnesses, when used with a handle and under re-enactment of the lead work, showed clear effects on the impulse; the Y-harness showed additional effects on the SL. Future studies should focus on the type of attachment of the harness, as well as the angle of attachment, which is altered by the size of the handler. The goal should be the development of harnesses adapted to the individual dog in order to subject these animals to as little stress as possible during their daily work.

## Figures and Tables

**Table 1:** Descriptive statistics of the measurement of the ground reaction forces in healthy guide dogs with different harnesses and collars.

		Condition						
**Parameter**	**Limb**	**C1 (Mean ± SD)**	**C2 (Mean ± SD)**	**C3 (Mean ± SD)**	**C4 (Mean ± SD)**	**C5 (Mean ± SD)**	**C6 (Mean ± SD)**	**C7 (Mean ± SD)**
pForce (%TF)	LF	29.52 ± 1.23	29.82 ± 1.16	29.28 ± 1.35	29.27 ± 1.23	29.57 ± 1.18	29.41 ± 1.26	29.38 ± 0.93
	RF	29.57 ± 1.14	29.99 ± 1.08	29.90 ± 1.33	29.82 ± 1.10	29.72 ± 1.23	29.64 ± 1.33	29.69 ± 1.32
	LH	20.67 ± 1.47	20.27 ± 1.42	20.27 ± 1.45	20.49 ± 1.45	20.57 ± 1.51	20.49 ± 1.53	20.49 ± 1.25
	RH	20.23 ± 0.75	19.92 ± 0.77	20.55 ± 1.20	20.43 ± 1.12	20.14 ± 0.91	20.47 ± 1.01	20.45 ± 1.02
TpForce (%SPD)	LF	30.40 ± 4.77 ^4,6,7^	31.23 ± 5.56 ^4,6,7^	33.87 ± 5.51 ^6,7^	36.10 ± 6.08 ^1,2,5^	31.31 ± 4.343 ^4,6,7^	41.75 ± 10.49 ^1,2,3,5^	41.83 ± 10.68 ^1,2,3,5^
	RF	31.18 ± 4.49 ^4^	31.26 ± 4.51	37.23 ± 10.39 ^5^	37.76 ± 0.52 ^5^	30.47 ± 4.10 ^3,4,6^	35.98 ± 8.06 ^5^	36.66 ± 10.95
	LH	24.45 ± 3.91	23.51 ± 3.43	24.68 ± 4.92	23.99 ± 3.71	23.42 ± 2.91	24.73 ± 4.60	24.33 ± 3.92
	RH	24.30 ± 3.10	23.19 ± 2.71	23.74 ± 2.68	23.74 ± 3.12	23.60 ± 2.55	23.88 ± 3.93	23.57 ± 2.37
vImpuls (%TF)	LF	31.89 ± 0.57 ^3,4,6,7^	32.13 ± 0.76 ^3,4,6,7^	30.89 ± 1.00 ^1,2,5^	30.87 ± 1.08 ^1,2,5^	31.90 ± 0.74 ^3,4,6,7^	30.93 ± 1.13 ^1,2,5^	31.16± 0.97 ^1,2,5^
	RF	31.77 ± 1.14	32.06 ± 0.92	31.41 ± 1.28	31.42 ± 1.20	31.87 ± 1.19	31.27 ± 1.23	31.29± 1.36
	LF	18.14 ± 0.95	17.94 ± 0.98	18.59 ± 1.14	18.75 ± 1.06	18.11 ± 1.00	18.74 ± 1.13	18.68± 1.07
	RH	18.20 ± 0.66 ^3,(4),6,(7)^	17.86 ± 0.57 ^3,4,6,7^	19.11 ± 0.89 ^1,2,5^	18.96 ± 1.14 ^(*1)*,2,5^	18.12 ± 0.60 ^3,4,6,7^	19.06 ± 1.21 ^1,2,5^	18.87± 0.99 ^(1),2,5^
SIpForce (%)	FL	1.19 ± 1.27	1.07 ± 0.87	1.32 ± 1.18	1.13 ± 0.97	1.16 ± 1.15	0.89 ± 0.96	1.18 ± 0.86
	HL	1.76 ± 1.63	1.68 ± 1.56	1.73 ± 0.90	2.04 ± 2.25	2.12 ± 1.99	1.38 ± 0.94	1.37 ± 1.21
SIvImpuls (%)	FL	1.10 ± 1.18	0.94 ± 0.81	1.31 ± 1.50	1.20 ± 1.31	1.57 ± 1.20	0.96 ± 0.71	1.64 ± 1.10
	HL	1.66 ± 0.84	1.28 ± 1.09	1.73 ± 0.88	1.56 ± 1.29	1.41 ± 1.16	1.66 ± 0.65	1.08 ± 1.03
SPD (%)	LF	26.23 ± 0.51	26.16 ± 0.45	25.96 ± 0.51	25.99 ± 0.47	26.14 ± 0.45	25.92 ± 0.60	26.02 ± 0.69
	RF	26.16 ± 0.55	26.28 ± 0.44	26.04 ± 0.63	26.17 ± 0.46	26.21 ± 0.60	26.07 ± 0.58	26.07 ± 0.65
	LH	23.60 ± 0.62	23.66 ± 0.46	23.86 ± 0.48	23.80 ± 0.43	23.66 ± 0.50	23.87 ± 0.43	23.88 ± 0.53
	RH	24.01 ± 0.38	23.89 ± 0.39	24.15 ± 0.44	24.05 ± 0.47	23.98 ± 0.40	24.15 ± 0.55	24.02 ± 0.48
SL (m)	LF	0.86 ± 0.04 ^6,7^	0.85 ± 0.04 ^6,7^	0.83 ± 0.04	0.83 ± 0.04	0.84 ± 0.04 ^6,7^	0.81 ± 0.04 ^1,2,5^	0.80± 0.04 ^1,2,5^
	RF	0.85 ± 0.05 ^6,7^	0.84 ± 0.03 ^7^	0.83 ± 0.05	0.82 ± 0.04	0.83 ± 0.04	0.81 ± 0.05 ^1^	0.81± 0.04 ^1,2^
	LH	0.85 ± 0.03 ^6,7^	0.85 ± 0.04 ^6,7^	0.83 ± 0.04	0.83 ± 0.05	0.85 ± 0.04 ^6,7^	0.81 ± 0.04 ^1,2,5^	0.81± 0.04 ^1,2,5^
	RH	0.85 ± 0.05 ^6,7^	0.84 ± 0.04 ^6,7^	0.83 ± 0.05	0.83 ± 0.05	0.84 ± 0.04 ^6,7^	0.81 ± 0.05 ^1,2,5^	0.81± 0.04 ^1,2,5^
v (m/s)	LF	1.15 ± 0.12	1.16 ± 0.09	1.11 ± 0.07	1.13 ± 0.09	1.16 ± 0.07	1.12 ± 0.11	1.09 ± 0.09
a (m/s²)	LF	0.00 ± 0.07	0.01 ± 0.04	0.02 ± 0.04	0.03 ± 0.05	0.04 ± 0.05	0.01 ± 0.03	0.00 ± 0.03

LF = left front limb, RF = right front limb, LH = left hind, RF = right hind, FL = front limbs, HL = hind limbs. C1 = collar and leash, C2 = usual working harness (Norwegian type) with leash, C3 = usual working harness with usual working handle (straight handle), C4 = usual working harness with curved handle, C5 = Y-harness with leash, C6 = Y-harness with straight handle, C7 = Y-harness with curved handle Superscript numbers denote significant differences between conditions, numbers in parentheses indicate a *p*-value of 0.06.. As an example: vImpuls (%TF) left front (limb 1) was significantly different in C3, C4, C6 and C7. The italicized numbers denote *p*-values just outside significance.

## Data Availability

The original contributions presented in the study are included in the article. Further inquiries can be directed to the corresponding author/s.
